# Agglomeration of particulate matter in chimneys using acoustic flow

**DOI:** 10.1016/j.heliyon.2024.e25306

**Published:** 2024-02-01

**Authors:** Kristina Kilikevičienė, Rimantas Kačianauskas, Vytautas Rimša, Artūras Kilikevičius

**Affiliations:** a Institute of Mechanical Science, Vilnius Gediminas Technical University, Vilnius, Lithuania; b Department of Aviation Technologies, Vilnius Gediminas Technical University, Vilnius, Lithuania

**Keywords:** Acoustic agglomeration, Silica microparticles, CFD, Relative entrainment factor

## Abstract

The emission of micrometer-sized particulate matter on an industrial scale is causing increasing environmental concern about air pollution. Numerous industries and research communities need help to reduce micrometer-sized pollutants in the atmosphere.

The current research investigates the acoustic agglomeration of particulate matter through a combination of experimental and numerical methods. Acoustic agglomeration is a process that involves using acoustic waves to influence the movement of particles in the air. Acoustic agglomeration operates by facilitating particle collision and simplifying the formation of agglomerates that are later removed through filtration.

This article is focused on research on acoustic pre-processing with the aim of reducing atmospheric pollution caused by toxic combustion products. The capture of fine silica particles with diameters ranging from 0.3 to 10 μm, emitted through the chimneys of industrial enterprises, can be considered a significant technological innovation.

The experimental part of the current research is conducted using a newly developed experimental bench. The assembly comprises the following key components: a wind tunnel, a particle dosing device, the agglomeration camera, and a particle concentration measurement device on the edge of the bench. A loudspeaker was used to evaluate the effect of sound pressure in the frequency range of 500–3000 Hz.

A comprehensive CFD study of the particles was conducted, which included analysis of the boundary layer, facilitating a better understanding of the behavior of the particles and its potential to agglomerate. An experimental study of particle agglomeration, using an acoustic field with a frequency range of 500–3000 Hz, demonstrated the effectiveness of particle agglomeration of different diameters. The efficacy of particle agglomeration is up to 80 % when the sound pressure values were 129–135 dB; the highest efficiency was found at excitation frequencies of 1500 and 3000 Hz, respectively.

## 1 Introduction

The increasing global population, the expansion of urbanization, and the increase of industrialization have led to a surge in air pollution caused by particulate matter, which presents a growing environmental problem. The impact of particulate matter on human health may vary depending on the type of particulate matter, its concentration, the duration of exposure, and the individual's susceptibility to contamination. It was also observed that the chemical composition of particulate matter varies due to strong absorption, as various toxic chemicals and microorganisms are absorbed. Thus, they become even more harmful to human health [1].

The distribution of particle mass [2] in small wood-fired boilers with stacks exhibits a bimodal size distribution (one peak between 0.12 and 0.32 μm and the other peak at 0.76 μm, therefore, it is believed that soot contributes to mode 0.76 μm). This could be explained because wood fuel has a higher concentration of lignin and a high concentration can increase the amount of soot. Unoxidized soot has the potential to accumulate and grow to a significant size. The particle mass size distribution in both new and old small biomass boilers exhibit a maximum value at an aerodynamic diameter of approx. 0.13 μm, which is consistent with other studies on biomass combustion devices [3]. Particles of various biomass fuels (bark, wood, and hydrolysis residue pellets) burnt in a 10 kW reactor under uniform conditions have a uniform size ranging from 0.02 to 0.7 μm [4]. In all cases of wood combustion, submicrometer particles (diameter less than 1 μm) accounted for more than 90 % of the PM mass in the range of 0.03–2.39 μm [2].

It has already been confirmed that the emissions of micrometer and submicrometer particulate matter from fossil fuel combustion exhaust pose a serious environmental pollution problem [[5], [6], [7], [8], [9]]. These particles, especially PM_2.5_, can remain suspended in the air for a long time and, as has been proven, pose a threat to human health, as they can carry various viruses and bacteria that can quickly enter the human body by breathing. Although many particle removal technologies have been developed, including electrostatic precipitators, high-efficiency filters, etc., these technologies are still ineffective in removing PM_2.5_. Therefore, developing an aerosol agglomeration method is crucial as it would induce coagulation among microparticles and improve the effectiveness of current particle removal technologies in removing PM_2.5_ [10].

One of the means of reducing the amount of small particle matter is acoustic agglomeration. Acoustic agglomeration is a process that involves using acoustic waves to influence particle movement in the air [[11], [12], [13], [14]]; thus facilitating particle collision and simplifying agglomerates (compounds of particles). As newly formed agglomerates continue to collide and merge, they trigger a cascade of particle growth. Acoustic agglomeration is considered to be the most efficient of the different particle transport mechanisms available. Acoustic agglomeration is an effective aerosol pretreatment technology that uses high-intensity sound to significantly increase the rate at which aerosol particles agglomerate before filtration [15]. Under optimized operating conditions, aerosol particulate number concentrations can be reduced by over 70 % in just a few seconds of residence time [[16], [17], [18], [19], [20]]. As a result, the size distribution of the aerosol particles shift toward larger diameter values. This technology has shown great potential to improve the effectiveness of dust collection filters. It can remove hazardous aerosols in the event of various accidents and dissipate mist in airports [21].

In recent decades extensive experimental studies have been carried out on the acoustic agglomeration of various aerosols. Studies analyzing coal-fired exhaust gases [22], variants of diesel fuel exhaust gas [[23], [24], [25]], flue gas [[26], [27], [28]] and liquid aerosols [23] have demonstrated sufficiently high agglomeration efficiency. The acoustic frequencies used in the literature vary widely, ranging from 46 Hz to 21 kHz. The optimal frequency is believed to be determined by the initial aerosol size distribution and tends to increase as the average particle size decreases. Frequencies between 1 and 3 kHz are favorable for micron and submicron-sized aerosol particles, such as coal-fired fly ash. However, ultrasound with a frequency greater than 10 kHz is more effective for exhaust gas from internal combustion engines containing nanometer and submicrometer-sized particles [[23], [24], [25]].

High-intensity acoustic fields can induce a process of particle agglomeration in suspended aerosols, known as acoustic or ultrasonic agglomeration. Various mechanisms for this process have been proposed. However, progress in understanding the agglomeration mechanisms necessary for optimizing acoustic agglomeration processes has been slow compared to experimental research. Many researchers have devoted significant attention to the study of acoustic agglomeration methods [[24], [25], [26], [27], [28], [29], [30], [31], [32]]. The important principles of acoustic agglomeration can be illustrated as follows: sound waves propagate through the aerosol, causing relative movement of particles and increasing the probability of collisions. Upon collision, smaller particles grow, and newly-formed particles continue to agglomerate with others, leading to the constant growth of aerosol particles. According to the literature, the most critical mechanisms of acoustic agglomeration are the orthokinetic collision mechanism and the hydrodynamic interaction. The orthokinetic interaction arises due to differences in the penetration speed of aerosol particles of various sizes into the sound field, leading to relative movement and collisions between the particles [33]. This interaction can vary due to the influence of the radiation background, acoustic flow, and turbulence. Being in the flow of particles of different diameters, the distance between which is approximately equal to the amplitude of the sound field displacement mechanism of orthokinetic type occurs when the particles move parallel to the direction of vibration from the source.

The actions of different forces in the flow and the inertial forces of the particles influence the difference in the amplitudes and phases of these objects, increasing the probability of collision and the agglomeration process. Another nature of occurrence of agglomeration is caused by hydrodynamic forces whose actions are directed at the particles. Acoustic fields form hydrodynamic forces, leading to the convergence of particles to a greater distance than the acoustic displacement created. In homogeneous particle-type flows, these forces change the relationship between the particles; therefore, the orthokinetic relationship is irrelevant because the acoustic field does not allow the particles to collide directly. The irregularity of the flow velocity distribution in the Oseen regime, called the acoustic trace effect, is one of the significant proofs of the hydrodynamic forces. Various processes involving particulate matter and its agglomeration, such as combustion, atomization, nano-powder conversion, granulation, and other types, are addressed to a greater extent using simulation processes [34,35]. In this way, the solution can provide a deeper understanding of the physical phenomena that occur. Combined Сomputational Fluid Dynamics (CFD) is based on solving the Navier-Stokes equations for fluid flow in motion [[36], [37], [38], [39], [40]]. The execution of numerical solutions can be performed as a particle-fluid interaction problem. In other words, the case where particles are solids that are immersed in a liquid medium can be considered.

In most cases, the region of the liquid medium is taken in a small space, within a distance equal to the distance between the particles, while the particles are separate boundaries of the region [41]. In the same way, problems with deformed particles, for example a droplet, can be solved [42]. If there is reciprocal coupling between fluid and particle flows, these flows are called bilaterally coupled. In this case, the particle motion can be changed by the flow, and the particle flow itself can affect the flow. This interaction can occur when the concentration of particles in the fluid being analyzed is high.

This article focuses on research on acoustic pretreatment for reducing atmospheric pollution caused by harmful combustion products. The experimental part of the current research is conducted using a newly developed experimental bench. The aim was to assess how sound pressure in the 500–3000 Hz frequency range affects the process of acoustic agglomeration. The experiments were carried out in a small-scale wind tunnel to investigate the efficiency of possible acoustic agglomeration. Numerical analysis includes CFD modeling of the particle motion. A comprehensive CFD study of particulate matter encompassing the boundary layer led to a deeper understanding of particle behavior and potential for agglomeration.

## 2 Methodology of numerical investigation

The main task of numerical investigation of particle agglomeration is to better understand particle or particle behavior in the acoustic field, particle size effect to its velocities and acting forces, which significantly influence particle agglomeration behavior. This approach helps to better understand the effects of agglomeration on the micro level, and it helps to establish more suitable parameters for practical agglomeration applications. For numerical investigation, software Ansys 2022 R1 CFX was used where the particle in the acoustic field was verified with the [43] test results. There are basic parameters of the acoustic field: air density *ρ*_*a*_ = 1279 kg/m^3^; viscosity *μ*_*m*_ = 0.0000183 μPa s; sound velocity *Us0* = 0.44 m/s; SPL = 136,1 dB; frequency *f* = 3000 Hz; angular frequency *ω* = 18,849,56 1/s; glass particles *ρ*_*p*_ = 2400 kg/m^3^.

The formula *U*_*f*_ = *Us0*sin (*wt*) defines the acoustic wave function, where the inlet and outlet have applied acoustic waves. The rest of the cube's walls have symmetry boundary conditions.

The accuracy of the numerical modeling results depends on the size of the object of study. Depending on the ratio between the size of the object itself and the geometric parameter, in this case the channel diameter, the influence of boundary conditions can vary significantly. For this purpose, it is important to determine the maximum size of the object, at which it will be possible to determine with the highest accuracy the natural and/or forced motion of particles in the acoustic field.

## 3 Results of acoustic agglomeration

### 3.1 Simulation results of acoustic agglomeration

The mesh strategy and its size effect significantly impact the accuracy of the numerical model.

Due to that, three mesh models were generated. Due to the high sensitivity of the mesh size to numerical results, the sensitivity study was carried out around a particle with a mesh size 2.7·10^−7^ m (nodes 51,400 elements 22,310) (1); 3.0·10^−7^ m (nodes 49,690, and elements 222,120) (2) and 3.3·10^−7^ m nodes 45,365, and elements 206,801 (3). The sensitivity study was designed to investigate the acting force and velocity of the particles in the acoustic field and validates the results with the analytical model.

(1) $$F_{dxp}=3\pi \rho_{m}\nu_{m}D_{p}\left(u_{mx}-u_{px}\right)$$

where: $\rho_{m}$ = 2400 kg/m^3^; $D_{p}$ = 8∙10^−6^ m; $u_{mx}-u_{px}$ – velocity difference between particles and fluid.

Numerical results show a minimal difference compared to well-known theoretical models ∼5 and 6 % of force and particle speed error, particle surfaces with 3·10^−7^ m size (2), which give acceptable calculation results, and this mesh model will be used for all calculations.

#### 3.1.1 Effect of particle size, its velocity, and acting forces

The particle size was investigated for a given particle size: *R* = 6; 8; 10 and 12 μm.

Calculations reveal that when the particle size is doubled, the velocity of the particles decreases approximately 4.6 times, and the acting forces of the particles increase approximately 1.8 times. These findings suggest that smaller particles, which experience higher motion in the acoustic field, are more prone to movement and thus can easily agglomerate into larger structures.

Relative entrainment factor calculations were performed using the formula below [44] to determine the frequencies of acoustic impact where the entrainment factor is highest for particles of varying diameters.

(2) $$q_{12}=\frac{\omega \left(\tau_{1}-\tau_{2}\right)}{\sqrt{\left(1+{\left(\omega \tau_{1}\right)}^{2}\right)\left(1+{\left(\omega \tau_{2}\right)}^{2}\right)}}$$

*ω -* Angular frequency $;\tau_{i}$ *-* Particle relaxation time $\tau_{i}=\rho_{i}D_{i}^{2}/18\mu_{m}\text{;}$ Particles diameter *D*_*p*_ and density *ρ*_*p*_**,** incompressible media (fluid) viscosity *μ*_*m*_.

**Fig. 1:**
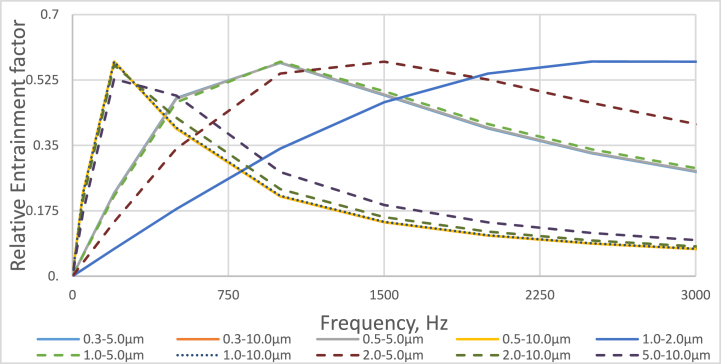
Results of the Relative Entrainment factor.

The results obtained by the relative tension factor (when evaluating particles with respective diameters of 0.3 with 5 and 10 μm; 0.5 with 5 and 10 μm; 1 with 2, 5 and 10 μm; 2 with 5 and 10 μm; 5 with 10 μm) are presented in Fig. 1.

Upon analyzing the Relative Entrainment factor results presented in Fig. 1, it can be concluded that the maximum factor values are achieved for frequencies up to 3000 Hz. Considering the Relative Entrainment factor values when the diameter of one particle is 10 μm the highest values range from 200 to 500 Hz (when the diameter of the second particle is 0.3, 0.5, 1, 2 and 5 μm); evaluating the factor values respectively when the diameter of one particle is 5 μm, the highest values range from 1000 to 1500 Hz (when the diameter of the second particle is 0.3, 0.5, 1 and 2 μm); evaluating the factor values respectively when the diameter of one of the particles is 2 μm, the highest values range from 2500 to 3000 Hz (when the diameter of the second particle is 1 μm).

**Fig. 2:**
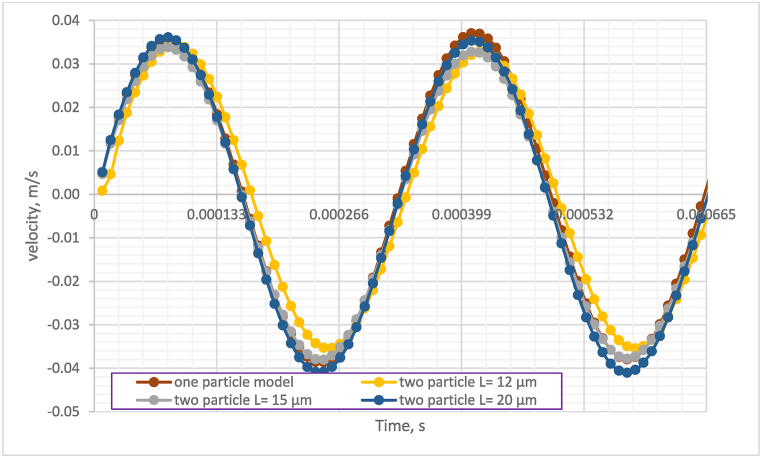
Sensitivity studies of two-particle interactions and the influence of the initial distance between their centers.

**Fig. 3:**
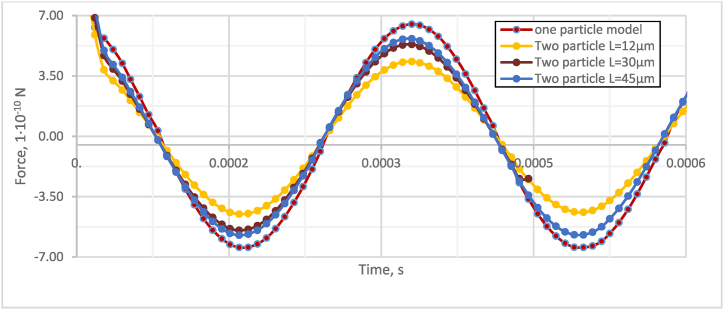
Sensitivity study of the force acting on particles in the acoustic field.

Investigating the interaction of two particles in the acoustic field is a complicated task, as outlined in Ref. [45]. However, this paper focuses solely on the agglomeration phenomena between two particles aligned with fluid flow. In reality, particles can be positioned at any distance and angle to the flow. Therefore, the development of an analytical equation for this physical model is challenging. As a result, the agglomeration of two particles is investigated in two main steps: the influence of distance on the agglomeration of two particles and the influence of the initial gap between two particles (Fig. 2, Fig. 3) and the direction of fluid flow, as demonstrated in the Supplementary Material (Fig. 8).

**Fig. 4:**
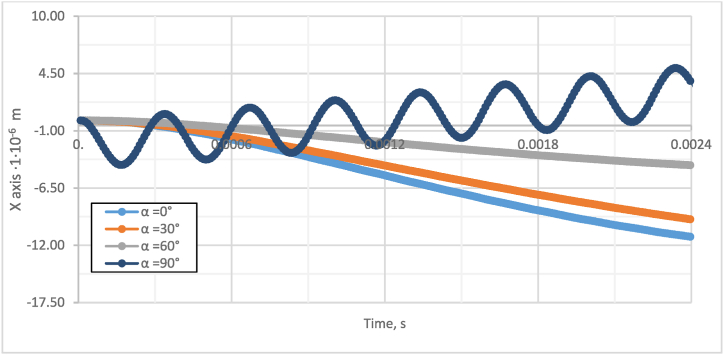
Investigation of the effect of the angle α between particles and fluid flow to the gap in the X-axis direction.

**Fig. 5:**
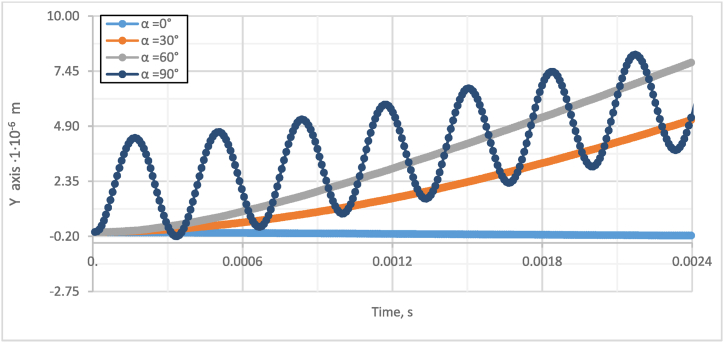
Investigation of the effect of the angle α between the particles and the fluid flow on the gap in the Y-axis direction.

Fig. 2 shows that increasing the initial distance between the particle centers from 12 to 20 μm results in a 14 % increase in the velocity of the particles. On the other hand, when particles are closer together (*L* = 12 μm), their velocity is slightly lower, and there is a 1 % delay in the maximum peak of particle speed compared to the two-particle model with *L* = 20 μm. This indicates that agglomeration decreases as the distance between particles increases. Moreover, decreasing the distance between the particles have a more significant effect on the acting particle forces. Specifically, Fig. 3 shows that when *L* increases from 12 μm to 20 μm, the acting forces drop by approximately 24 %. Investigation of the effect of the angle α between particles and fluid flow to the gap in the direction of X and Y axles (Fig. 4, Fig. 5).

**Fig. 6:**
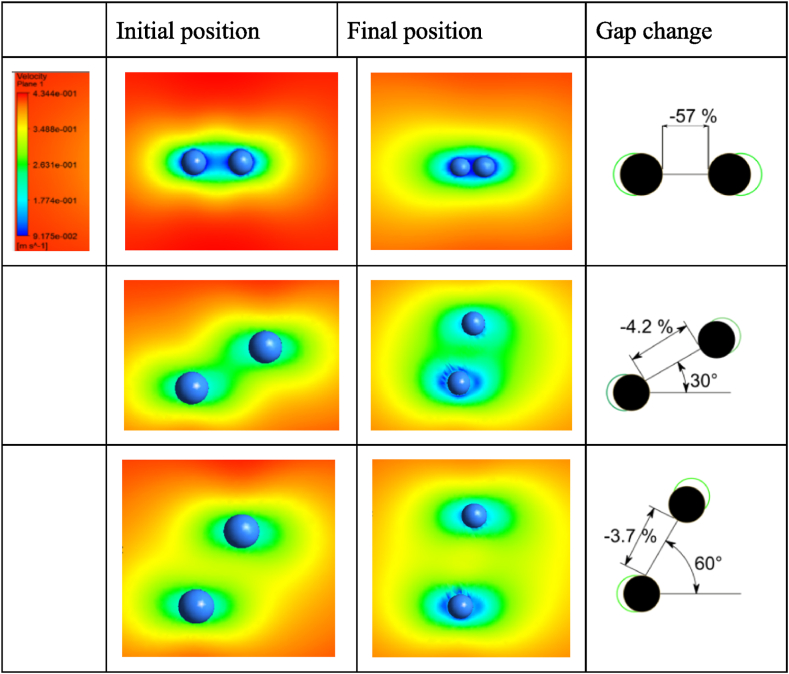
Sensitivity study of initial particle angle α effect on particle agglomeration.

As could be observed from Figs. 4 and 5, the smaller the initial angle between the particles, the smaller the gap between the particles achieved through the same period. Moreover, on the contrary, with a larger initial angle, a more significant displacement of the particle along the Y-axis is achieved. When the particles are perpendicular to the flow of the fluid, they no longer approach each other, but there is a visible undulation of the particles, and they move away from each other. The sensitivity study of the effect of the initial particle angle α effect on the agglomeration of particles is presented in Fig. 6.

A numerical investigation reveals that the initial angle α significantly impacts particle agglomeration Fig. 6. When the particles align with the acoustic flow, the gap decreases by up to 57 % in 2∙10^−3^s seconds. However, if the particles are not aligned in one line, the gap decreases by 4.2 % or 3.7 % for angles α of 30° and 60°, respectively.

## 4 Results of experimental acoustic agglomeration

While conducting the theoretical research, it was set that in the range of particle diameters from 0.3 to 10 μm, the maximum Relative Entrainment factor is in the frequency range from 250 to 3000 Hz.

The current study aims to demonstrate that particulate matter in the air could be pre-enlarged by acoustic agglomeration, thereby facilitating its subsequent removal via air filtration in chimney systems. The experiments were carried out in a small-scale wind tunnel to investigate the possible effectiveness of acoustic agglomeration.

**Fig. 7:**
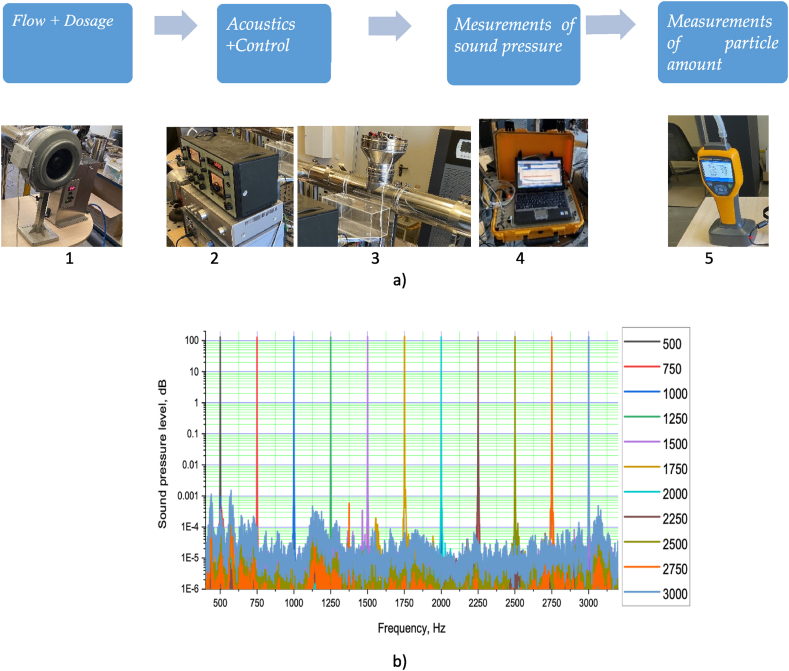
Diagram representing the experiment bench when the loudspeaker is used to generate sound pressure (a) and the results of sound pressure measurements (b).

The experiment bench simulates airborne solid particulate matter in the system (similar to a stack element) with pre-acoustic particulate agglomeration occurring before particle measurement. It is shown in Fig. 7a, the wind tunnel, sound pressure measurement equipment (Bruel&Kjær data processing system 9727), a particle dosing device (Pallas RGB100), the agglomeration zone and the particle measurement device at the far end of the wind tunnel are the key components of the assembly.

**Fig. 8:**
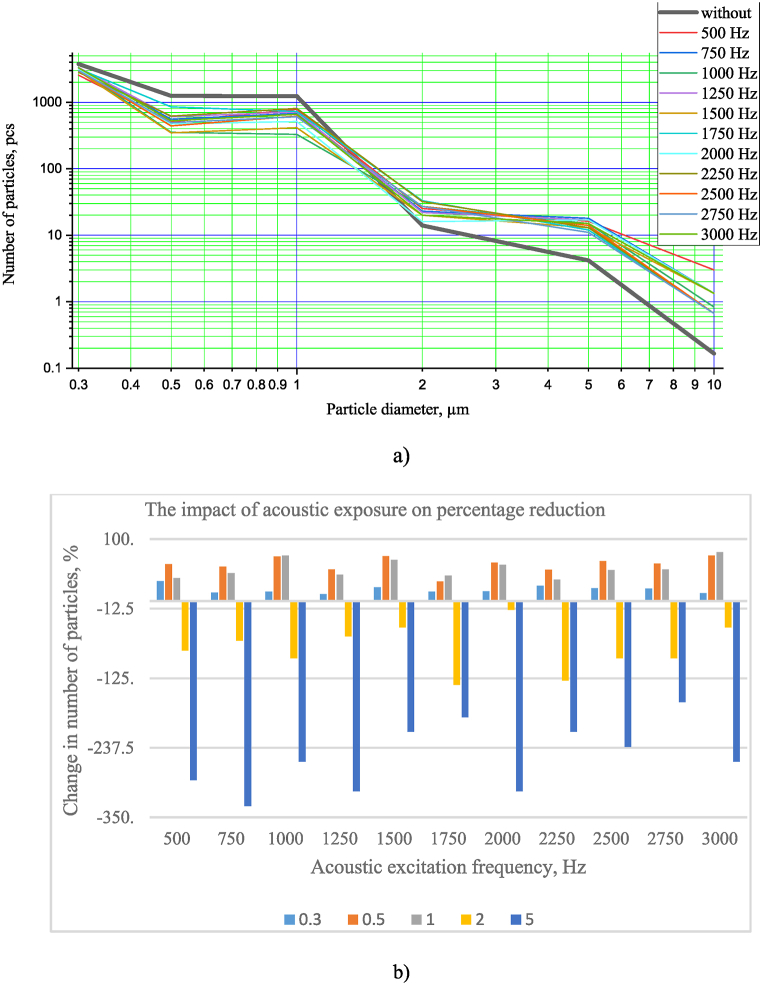
Results of particle number measurements with and without acoustic effects (a), results indicating the percentage change in the particulate matter under acoustics (b).

Test particles with a primary component of silica (ISO 12103-1 A1 ultra-fine test dust) are introduced upstream of the agglomeration zone. The polydispersity characteristics of the test particles may be typical of airborne PM.

The concentration of test particles was selected to closely match the concentration of particles typically found in such cases to simulate the particles in a wood-burning chimney system. A Fluke 985 six-channel particle counter equipped with an isokinetic sampling probe (5 positions in Fig. 7) to determine the numerical concentration of particulate matter. The particle size range of the device is (0.3, 0.5, 1.0, 2.0, 5.0 and 10.0) μm. The sample flow rate is 2.83 L/min. The tests were conducted in the cumulative mode. Before each measurement, the instrument was calibrated using a zero-filter check to ensure the accuracy of no more than one particle per 5 sampling periods, per JIS 89921. The sampling location is outside of the agglomeration area. The acoustic agglomeration zone consists of a high-frequency loudspeaker, an amplifier, and a signal generator.

The studies investigate the change of PM concentration after purification using acoustic agglomeration as a pre-treatment of the flow, as well as without its use. To avoid and reduce uncertainties for the generation of the particle source, each study consists of six repetitions, with a calculation of their mean value. The sound level was maintained at a certain level and the PM measurements were made for 15 s. The total concentration of particles in the flow was much higher than the losses of particles deposited on the walls of the booth, so the error can be neglected. The effect due to acoustic agglomeration is much more important than the available flow turbulence and secondary nonlinear effects at low acoustic intensities. Each experiment begins with recording the flow velocity and temperature. The test bench section is cleaned of possible particle deposits and blown out at the end of each measurement. The frequency of the harmonic signal set in the generator during the experiment is 500 Hz; the flow rate is kept equal to 0.2 m/s, which is regulated by the frequency converter. In the first stage, the concentration of PM without acoustic influence that is not in the agglomeration zone is investigated. In the second stage, the acoustic installation is started and PM measurements are made outside the agglomeration zone. This procedure is repeated to investigate other frequencies of acoustic influence. Sound pressure measurements were conducted at various frequencies: 500, 750, 1000, 1250, 1500, 1750, 2000, 2250, 2500, 2750 and 3000 Hz. The results of the sound pressure measurement obtained at the corresponding excitation frequencies are presented in Fig. 7b, indicating that the sound pressure values ranged from 129 to 135 dB.

The effects of acoustics on PM agglomeration are determined primarily by sampling downstream of the agglomeration zone. As shown in Fig. 8a, b, the experimental results with and without acoustics exhibit 6 % and 8 % errors, respectively. Additionally, a loudspeaker was used to investigate the influence of sound pressure in the range of 500–3000 Hz. An image of the experimental bench is presented in Fig. 7 (3 position). The generator and amplifier (2 position in Fig. 7) were utilized to produce the sound pressure.

On analyzing the results presented in Fig. 8a, b, it was observed that changes in the particle concentration could be observed across all excitation frequencies. These changes reveal that the effect of acoustics reduces the number of particles up to 1 μm; respectively, the effect of acoustics increases the number of particles from 2.0 μm in diameter. When evaluating the change in the number of particles by diameter, it was discovered that the impact of acoustics results in a reduction in the number of particles with a diameter of up to 1 μm, ranging from 11.4 to 32.2 % (considering the particulate matter of 0.3 μm in diameter), ranging from 31.5 to 73.7 % (considering the particulate matter of 0.5 μm in diameter), and ranging from 34.6 to 79.0 % (considering the particulate matter of 1.0 μm in diameter). Consequently, it was determined that the effect of acoustics increases the number of particles with a diameter of 2.0 μm from 14.3 to 135.7 % (considering particles with a diameter of 2.0 μm) and from 163.8 to 331.7 % (considering particles with a diameter of 5.0 μm). It was also revealed that the most significant decrease in particles, up to 1.0 μm in diameter, was observed at 500 Hz (considering the particulate matter with a diameter of 0.3 μm), 1000 and 1500 Hz (considering the particulate matter with a diameter of 1.0 μm).

**Fig. 9:**
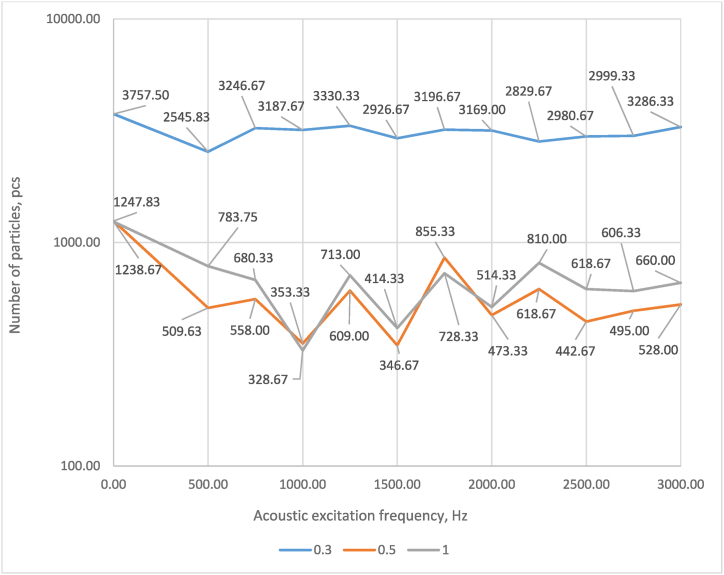
Relationship between the change in the number of particles with diameters of 0.3, 0.5 and 1.0 μm and the acoustic wake frequency.

Fig. 9 presents the relationship between the change in the number of particles with diameters of 0.3, 0.5 and 1.0 μm and the acoustic wake frequency.

The results demonstrated in Fig. 9 are similar to those obtained from the Relative Entertainment factor and presented in Fig. 1. It was set that the lowest number of particles with a diameter of 0.3 μm was obtained at a frequency of 500 Hz. In comparison, the smallest particles with a diameter of 0.5 μm and 1.0 μm were obtained at 1000 Hz and 1500 Hz, respectively. These results reveal that the agglomeration may vary slightly depending on the different frequencies of the sound pressure; therefore, further experimental studies will be conducted with different excitation frequencies to investigate this further.

## 5 Conclusions

Evaluation of the change in the number of particles revealed that the effect of acoustics reduces the number of particles up to 1 μm in diameter; specifically, for particles with a diameter of 0.3 μm, the reduction was from 11.4 % to 32.2 %; for particles with a diameter of 0.5 μm – from 31.5 to 73.7 %; for particles with a diameter of 1.0 μm – from 34.6 to 79.0 %. Respectively, evaluating the number of particles with a diameter of 2.0 μm and greater, it was established that the acoustic effect increases the number of particles with a diameter of 2.0 μm - from 14.3 to 135.7 % and with a diameter of 5.0 μm - from 163.8 to 331.7 %. When evaluating the impact of the frequency of acoustic exposure, it was observed that the highest reduction in the number of particles with diameters up to 1.0 μm occurred at 500 Hz (for particles of 0.3 μm) and 1000 and 1500 Hz (for particles of 0.5 and 1.0 μm), respectively.

A numerical simulation has confirmed that particle agglomeration can be effectively modeled using the finite-volume method, resulting in a low numerical error of around 4 % compared to the analytical Stokes model. The analytical model is highly complex when considering multi-particle interactions at various angles, sizes, and positions.

The speed of particle motion decreases by 10 % in a two-particle model and up to 60 % in a three-particle model. This causes the particle approach to increase by up to 52 % in a two-particle model when aligned with the acoustic flow. In contrast, in a three-particle model, this value is only around 10 % because the particles are shadowed by each other.

## Data availability

Data will be made available on request.

## Funding

This research received no external funding.

## CRediT authorship contribution statement

**Kristina Kilikevičienė:** Writing – original draft, Methodology, Investigation, Data curation, Conceptualization. **Rimantas Kačianauskas:** Writing – review & editing, Supervision, Methodology. **Vytautas Rimša:** Writing – original draft, Software, Methodology, Conceptualization. **Artūras Kilikevičius:** Writing – review & editing, Validation, Supervision, Methodology, Investigation.

## Declaration of competing interest

The authors declare no conflicts of interest.
